# An Australian Indigenous community-led suicide intervention skills training program: community consultation findings

**DOI:** 10.1186/s12888-017-1380-5

**Published:** 2017-06-13

**Authors:** Bushra Nasir, Steve Kisely, Leanne Hides, Geetha Ranmuthugala, Sharon Brennan-Olsen, Geoffrey C. Nicholson, Neeraj S. Gill, Noel Hayman, Srinivas Kondalsamy-Chennakesavan, Maree Toombs

**Affiliations:** 1 Rural Clinical School, Faculty of Medicine, The University of Queensland, Toowoomba, QLD Australia; 2 Departments of Psychiatry, Community Health & Epidemiology, Dalhouise University, Halifax, Canada; 3 School of Psychology, Faculty of Health and Behavioural Sciences, The University of Queensland, Brisbane, QLD Australia; 4 School of Rural Medicine, University of New England, Armidale, NSW Australia; 5 Australian Institute for Musculoskeletal Science (AIMSS), The University of Melbourne, Melbourne, Australia

**Keywords:** Indigenous, Community-consultation, Suicide prevention, Suicide intervention

## Abstract

**Background:**

Little is known of the appropriateness of existing gatekeeper suicide prevention programs for Indigenous communities. Despite the high rates of Indigenous suicide in Australia, especially among Indigenous youth, it is unclear how effective existing suicide prevention programs are in providing appropriate management of Indigenous people at risk of suicide.

**Methods:**

In-depth, semi-structured interviews and focus groups were conducted with Indigenous communities in rural and regional areas of Southern Queensland. Thematic analysis was performed on the gathered information.

**Results:**

Existing programs were time-intensive and included content irrelevant to Indigenous people. There was inconsistency in the content and delivery of gatekeeper training. Programs were also not sustainable for rural and regional Indigenous communities.

**Conclusions:**

Appropriate programs should be practical, relevant, and sustainable across all Indigenous communities, with a focus on the social, emotional, cultural and spiritual underpinnings of community wellbeing. Programs need to be developed in thorough consultation with Indigenous communities. Indigenous-led suicide intervention training programs are needed to mitigate the increasing rates of suicide experienced by Indigenous peoples living in rural and remote locations.

## Background

Indigenous Australians have twice the suicide rate of non-Indigenous Australians; and the rates are four times higher among Indigenous youth [1, 2]. Suicide rates also tend to increase with remoteness so that Indigenous populations in very remote areas have twice the suicide rate as those in major cities [3]. This is of concern given Indigenous Australians comprise a large proportion of the population of very remote and remote residents in Australia [3].

Despite the high burden of suicide amongst Indigenous Australians there are relatively few Indigenous-specific suicide prevention programs available [4]. Indigenous communities share elements of culture, language and heritage, and it is essential that suicide prevention and intervention programs encourage connectedness, facilitate assimilation, and maintain cultural heritage [5]. Reports have shown that a community-led participatory approach is required to develop a comprehensive, effective and long-term program that can tackle the multi-dimensional aspects of suicide prevention and intervention [6].

### Gatekeeper training

Gatekeeper suicide prevention training teaches specific groups of people the knowledge, attitudes and skills necessary to identify people at risk of suicide and implement appropriate preventive interventions [7]. Frontline gatekeeper education and intervention training is one of the most effective suicide prevention strategies available [7, 8]. International studies have shown that gatekeeper training programs can contribute to significant improvements in trainer skills in suicide prevention [9–13]. However, other research has questioned the use of Western gatekeeper training programs as an effective suicide prevention tool for Indigenous communities [14, 15]. There is therefore a need for a culturally tailored, diverse, and systematic suicide prevention program targeting Australian Indigenous people at risk of suicide. An effective early intervention program aimed at increasing gatekeepers’ ability to identify suicide risk and implement effective suicide prevention strategies could enable Indigenous communities to maintain sustainable suicide prevention programs [9, 10].

### The Indigenous Network Suicide Intervention Skills Training project

The Indigenous Network Suicide Intervention Skills Training (INSIST) project aims to design, implement and evaluate a new multi-faceted, culturally-tailored intervention training program for preventing suicide among Indigenous people, especially Indigenous youth. The project has three aims: 1) to increase the knowledge and awareness of suicide risk factors; 2) develop a sense of connectedness among gatekeepers, at-risk groups, health services and support groups; and 3) to develop knowledge and awareness of professional and ethical responsibilities related to suicide risk among Indigenous communities.

The first phase of the project was to develop an intervention training package, drawing on existing models, through community based participatory research. This paper reports the findings from consultations held with community members and key stakeholders for the purpose of a) reviewing existing Indigenous gatekeeper training packages; b) assessing cultural appropriateness of the content in the programs; and c) identifying the key components of a culturally appropriate gatekeeper training program.

## Methods

### Research approach

Community based participatory research (CBPR), in which consultations are held with relevant parties and organisations as well as members of the broader community [16], is highly beneficial for addressing complex issues, particularly in Indigenous communities [17, 18]. We undertook such consultations in Indigenous communities in rural and regional Southern Queensland, Australia using an approach informed by guidelines from the Consolidated Criteria for Reporting Qualitative Research (COREQ) [19] and further explained by the Standards for Reporting Qualitative Research (SRQR) [20].

Involvement of Indigenous researchers, as well as participation of Indigenous community members and organisations were critical to the study. Indigenous as well as non-Indigenous community members, stakeholders and service providers all played a key role in the consultations. Consultations involved both one-on-one interviews, as well as focus groups with larger groups of people. Detailed participant demographics were not recorded to maintain confidentiality. However, consultations were carried out with both non-Indigenous and Indigenous people, and people representative from all demographic backgrounds of the consultation settings. Community concerns were prioritised, and cultural sensitivity and relevance was maintained during the consultation due to the nature of the sensitive topic of discussion. The resulting outcomes of each consultation were interpreted and discussed with participants to ensure the validity of the generated outcomes.

### Data analysis

Qualitative data were generated from unidentified notes taken during in-depth, semi-structured consultations allowing for themes and ideas to be relayed, and the discovery of elaborate information important to participants. Questions explored the current perceptions of gatekeeper training programs for Indigenous people, especially youth; i.e. “*Can you comment on the cultural appropriateness of current suicide prevention training programs?*” and “*Can you tell me about some components that would be beneficial for suicide prevention and intervention as part of a culturally appropriate gatekeeper training program*?”

On completion of each consultation, discussion with community members and investigators identified significant ideas and observations that were considered fundamental to the consultation process; upon reaching consensus, outcomes were documented. The community members played a key role in checking the validity and relevance of the resulting outcomes and consultation themes. Indigenous communities and organisations were provided the opportunity to ‘own’ the date collected and given a chance to ‘voice’ their concerns. Anonymous, systematic, thematic analysis was then performed with the information gained from community. Quality analysis assessment [19, 20] was conducted by the investigators to enhance and substantiate the analysis outcomes.

## Results

### Study sample

Initially, the research team spent time building rapport with communities where suicide prevalence was known to be high. This led to consultations with multiple community members, health care service providers, volunteer workers, and key organisations providing mental health as well as physical health support programs (Table 1). These frontline service providers were deemed to be most at need for training in the identification of at risk individuals and suicide prevention. A total of 29 consultations were conducted that ranged in duration from 10 min to 2 h; some consolations were conducted collectively with groups of people and therefore took longer than individual consultations.

**Table 1:** List of key organisations from which representatives were consulted

Name of organisation	Location	ASGS-RA^a^
Charleville & Western Areas Aboriginal & Torres Strait Islanders Corp for Health (CWAATSICH)	Roma	RA 3
QAS - Queensland Ambulance Service	Roma	RA 3
Queensland Department of Allied Health Services	Roma	RA 3
Maranoa Regional Council (Community Support/Youth)	Roma	RA 3
Standby Response Services	Roma	RA 3
Gateway To Training	Goondiwindi	RA 3
DRUGARM	Goondiwindi	RA 3
Centre Care Toowoomba –Partners in Recovery (PIR)	St George	RA 4
St. George Aboriginal Housing Company	St George	RA 4
QPS-Queensland Police Service	St George	RA 4
RFDS - Royal Flying Doctor Service	St George	RA 4
Charleville & Western Areas Aboriginal & Torres Strait Islanders Corp for Health (CWAATSICH)	Charleville	RA 5
Community Allied Health Services	Charleville	RA 5
QPS - Queensland Police Service	Charleville	RA 5
Charleville Neighbourhood Centre	Charleville	RA 5
QAS –Queensland Ambulance Service	Charleville	RA 5
Far West Family Violence	Charleville	RA 5
Royal Flying Doctor Service	Charleville	RA 5
Lifeline Darling Downs	Charleville	RA 5
PHaMs –Personal Helpers and Mentors	Charleville	RA 5
QPS - Queensland Police Service	Charleville	RA 5
Queensland Health- HOPE Project	Charleville	RA 5
Centre Care Toowoomba- Domestic & Family Violence	Cunnamulla	RA 5
Centacare Toowoomba –Partners in Recovery (PIR)Far West Family Violence Service	Cunnamulla	RA 5
Paroo Regional Council	Cunnamulla	RA 5
QPS - Queensland Police Service	Cunnamulla	RA 5
Royal Flying Doctor Service	Cunnamulla	RA 5
PHaMs –Personal Helpers and Mentors Service	Cunnamulla	RA 5
Cunnamulla Aboriginal Corporation for Health-Primary Health Centre (CACH)	Cunnamulla	RA 5
Queensland Country Practice Rural Remote Medical support-Cunnamulla Medical Clinic	Cunnamulla	RA 5

^a^ASGS-RA: Australian Statistical Geography Standard - Remoteness Area [25]

### Review of existing models of Indigenous gatekeeper training packages

All participants had previously undertaken generic gatekeeper suicide prevention training or had detailed knowledge in the area. Participants highlighted the problematic nature of the current delivery of gatekeeper training programs by a number of entities funded under different schemes, without any consistency. The majority indicated that existing gatekeeper trainings were “*lengthy and contained irrelevant information for Indigenous people”.* This was considered “*burdensome*”, given the limited resources available in regional areas. They reported many people were “*unaware of the services available”*, which often resulted in suicidal people “*not accessing the help available and the local communities trying to manage the risk of suicide themselves”*. There was a strong desire for community appropriate training, and supporting tools to empower and sustain the training provided. Participants indicated that they needed “*something that they can use straight away and don’t need to spend too much time learning or that is too hard for anyone.”*

### Identification of culturally inappropriate content in existing gatekeeper programs

Participants considered existing models were not culturally tailored to local Indigenous communities and indicated that they “*don’t like what is currently out there as it doesn’t really work”.* Shortfalls included the lack of appropriate language and the use of culturally irrelevant scenarios. “*No one can really understand them and know how to use it in real life.”* Communities indicated there was still a sense of “*shame*” when talking about suicide related issues. It was acknowledged that current training and knowledge within the community for identifying and preventing suicide was very low, due to gaps in training packages. Furthermore, it was indicated that little consultation with Indigenous communities was carried out and cultural training was often not provided to the trainers.. “*They (the trainers) don’t really know our culture.”* Current models for suicide prevention were deemed to be “*too lengthy for busy staff and too expensive*”, which deterred communities from accepting these training packages as appropriate or useful. It was also recognized that existing models were not sustainable within these rural and regional communities, due to the lack of staff and resources, and “*socially unacceptable content”* surrounding suicide for Indigenous communities.

### Key components required for a culturally appropriate Indigenous gatekeeper training program

As a result of these extensive community consultations, several key components of culturally appropriate gatekeeper training programs were identified (Table 2). Foremost was the need for a long-term sustainable program that enables local Indigenous communities to manage suicide risk. Participants highlighted a need for “*culturally acceptable, practical, easily implemented and self-sustainable*” gatekeeper training programs. “*We need something now, and something that we can easily use and keep our people safe”.* They suggested the programs use the local Indigenous language and relevant, relatable scenarios to deliver the training in a more effective method*. “It has to be for us so we can use it properly and something we can relate to”.* Supporting technology, such as a mobile app would also help gatekeepers to receive relevant updates. Participants indicated that “*we need to learn the technology to make things quicker and use it to help us make safer communities.”*

**Table 2:** Key components required for a culturally appropriate gatekeeper training package

• Short duration	
• Practical – easy to use	
• Relevant – language & scenarios	
• Sustainable – self-empowering communities	
• Generalisable – adaptable across all Indigenous communities	
• Inexpensive – available for everyone	
• Integration of current valuable resources	
• Focus on social, emotional, cultural and spiritual underpinnings of community wellbeing	

Additionally, it was considered that an Indigenous-specific gatekeeper training program should have generalisability to various Indigenous communities in Australia. “*Not all communities have the same culture, we have different languages and cultures all across the state and country.”* A focus on the social, emotional, and spiritual underpinnings of community wellbeing, not just cultural, was also emphasised and deemed necessary for an effective suicide prevention training package. “*We need to have the healing and spiritual context included and we want to have them understand what this means for us in our lives.”* Lastly, it was suggested that the effective components and resources of existing training programs and resources could be integrated into a new culturally appropriate gatekeeper training program. *“We really need an Indigenous appropriate training program otherwise there won’t be much change.”*

## Discussion

Our study found a lack of community-led suicide intervention training programs that could be effective in Indigenous communities. Previous research has also highlighted the lack of Indigenous-appropriate training programs [21]. Although previous studies have adapted various forms of culturally-modified gatekeeper training programs [9, 10, 12], they did not use CBPR to develop an Indigenous specific training program that meets community needs. We also found general agreement among participants on the limitations of existing programs and on the need for a culturally appropriate suicide prevention program across all of rural and remote Queensland. A need for systematic evaluation of the cultural appropriateness and effectiveness of Indigenous suicide prevention programs was also highlighted.

Given that suicide occurs after a sequence of events and decisions, prevention should be based on understanding the individual, and the decisions they are making [22]. Intervention strategies with high levels of local Indigenous community involvement are the most effective [13] but also require communities to become self-sufficient and able to both recognize risk factors and implement early intervention. To do this, a culturally-designed, systematic and consolidated Indigenous gatekeeper training program is imperative [21]. Supporting tools using technology [23, 24], may also be beneficial for gatekeepers.

No adolescents (<18 year olds) were consulted at this stage of the community consultation, which may be a limitation. Due to the nature of the consultations, talking to adolescents about suicide, suicide prevention and interventions had complex ethical and practical issues and hence, anyone younger than 18 years of age were not included for consultations at this stage.

## Conclusion

Existing models of Indigenous gatekeeper training and other suicide prevention models are not culturally tailored and do not empower Indigenous peoples. Practical suicide prevention programs are needed to mitigate the increasing rates of suicide experienced by Indigenous peoples living in rural and remote locations. Developing such programs requires a community-led approach to identify gaps in models developed for mainstream populations and to identify key elements that would make the program specific to Indigenous communities.

## Note

We respectfully identify Aboriginal and Torres Strait Islander people as Indigenous Australians within this manuscript.
